# Prevalence and Predictors of High Blood Pressure Among Women of Reproductive Age and Children Aged 10 to 14 Years in Guatemala

**DOI:** 10.5888/pcd17.190403

**Published:** 2020-07-23

**Authors:** Cassandra M. Pickens, Rafael Flores-Ayala, O. Yaw Addo, Ralph D. Whitehead, Mireya Palmieri, Manuel Ramirez-Zea, Yuling Hong, Maria Elena Jefferds

**Affiliations:** 1 Epidemic Intelligence Service Program, Center for Surveillance, Epidemiology, and Laboratory Services, Centers for Disease Control and Prevention, Atlanta, Georgia; 2 Division of Nutrition, Physical Activity, and Obesity, National Center for Chronic Disease Prevention and Health Promotion, Centers for Disease Control and Prevention, Atlanta, Georgia; 3 Emory University Global Health Institute, Atlanta, Georgia; 4 McKing Consulting Corporation, Atlanta, Georgia; 5 Nutrition and Micronutrients Unit, Institute of Nutrition of Central America and Panama (INCAP), Guatemala City, Guatemala; 6 INCAP Research Center for the Prevention of Chronic Diseases, Institute of Nutrition of Central America and Panama, Guatemala City, Guatemala; 7 Division for Heart Disease and Stroke Prevention, National Center for Chronic Disease Prevention and Health Promotion, Centers for Disease Control and Prevention, Atlanta, Georgia

## Abstract

**Introduction:**

Data on the prevalence and predictors of high blood pressure among children and non-pregnant women of reproductive age are sparse in Guatemala. Our objective was to identify the prevalence and predictors of high blood pressure among women of reproductive age and children in Guatemala.

**Methods:**

We analyzed data on blood pressure among 560 children aged 10 to 14 years and 1,182 non-pregnant women aged 15 to 49 from a cross-sectional, nationally representative household survey, SIVESNU (Sistema de Vigilancia Epidemiológica de Salud y Nutrición). We defined high blood pressure among children by using 2004 and 2017 US pediatric guidelines. We defined high blood pressure among women by using 1999 World Health Organization (WHO) and 2017 American College of Cardiology/American Heart Association (ACC/AHA) guidelines. We used multivariable logistic regression to identify significant predictors of high blood pressure. A base model included key covariates (age, ethnicity, socioeconomic index, anthropometric indicators) and accounted for complex sampling. We used backward elimination to identify additional candidate predictor variables.

**Results:**

High blood pressure was prevalent among 8.0% (95% confidence interval [CI], 5.4%–10.7%) and 14.0% (95% CI, 10.6%–17.5%) of children using 2004 and 2017 guidelines, respectively; and among 12.7% (95% CI, 10.7%–14.8%) and 41.1% (95% CI, 37.7%–44.4%) of women using 1999 WHO and 2017 ACC/AHA guidelines, respectively. Levels of awareness, treatment, and control of high blood pressure were low in women. Among children, significant predictors of high blood pressure were obesity, overweight, and indigenous ethnicity. Among women, significant predictors of high blood pressure included obesity, overweight, and diabetes.

**Conclusion:**

The prevalence of high blood pressure was high among Guatemalan women and children. Overweight and obesity were strong risk factors for high blood pressure. Increasing obesity prevention and control programs may help prevent high blood pressure, and expanding high blood pressure screening and treatment could increase awareness and control of high blood pressure in Guatemala.

**Summary**

**What is already known on this topic?**

Latin America is going through a nutrition transition, in which rates of overnutrition and noncommunicable diseases are increasing.

**What is added by this report?**

The prevalence of high blood pressure was high in a national sample of children aged 10 to 14 years and non-pregnant women aged 15 to 49 in Guatemala. Overweight and obesity were the strongest risk factors for high blood pressure identified thus far among these populations.

**What are the implications for public health practice?**

Obesity prevention and control programs might help prevent high blood pressure in children and reproductive-aged women in Guatemala.

## Introduction

High blood pressure is a leading cause of death and disability worldwide (1). National data on the prevalence and predictors of high blood pressure can be used to design health policies and programs and to track progress toward national health targets. National data on high blood pressure are particularly needed in countries such as Guatemala that are undergoing a nutrition transition characterized by increased consumption of processed foods (2). Several recent studies highlighted the prevalence and predictors of high blood pressure in Latin America (3–6). However, nationally representative information is lacking in Guatemala. Data on chronic diseases and their risk factors are particularly sparse among women and children in Guatemala.

The objective of our study was to describe the prevalence and predictors of high blood pressure among Guatemalan children aged 10 to 14 years and non-pregnant Guatemalan women of reproductive age (15–49) using national data from the 2017 Sistema de Vigilancia Epidemiológica de Salud y Nutrición (SIVESNU) (Epidemiological Health and Nutrition Surveillance System). We hypothesized that overweight and obesity would be strong risk factors for high blood pressure (7,8).

## Methods

SIVESNU is an annual, nationally representative, cross-sectional, continuous household survey in Guatemala. Primary objectives of the 2017 SIVESNU included assessing the prevalence of overweight, obesity, and anemia among women and children. SIVESNU is administered by the Guatemalan Ministry of Public Health and Social Assistance, with support from the Institute of Nutrition of Central America and Panama (INCAP), the United Nations Children’s Fund, the US Agency for International Development, and the US Centers for Disease Control and Prevention (9).

SIVESNU uses multistage sampling. First, in SIVESNU 2017, 100 enumeration areas were selected by using probability proportional to size sampling from a nationally representative sampling frame. Cartographers mapped all households in selected enumeration areas. Second, 30 households were randomly selected from each enumeration area. Households were visited up to 3 times. People residing in the household were eligible to participate; up to 1 woman aged 15 to 49 and 1 school-aged child were randomly selected per household. People who refused were not replaced, nor were households without eligible participants (10). Sample size calculations for SIVESNU 2017 were based on having 80% power to estimate annual changes in the prevalence in overweight/obesity and anemia among non-pregnant women aged 15 to 49. An expected 2,152 non-pregnant women aged 15 to 49 would be selected from 3,000 recruited households, accounting for household demographics and nonresponse (10). The 2017 sample had approximately 80% power to estimate a 7.0% annual change in the prevalence of overweight/obesity (10).

### Data collection

Trained enumerators administered surveys in Spanish or, via interpreter, in a local indigenous language, using validated instruments. Any household member aged 18 years or older could respond to the household questionnaire. We derived a socioeconomic index variable by using principal components analysis with 5 household-level input variables (radio, television, toilet, exclusive cooking area, and handwashing soap). Household food security was assessed by using the Latin American and Caribbean Food Security Scale (11). Up to 1 woman aged 15 to 49 per household responded to the women’s questionnaire, and up to 1 school-aged child per household responded to the children’s questionnaire. We restricted our analysis of children to those aged 10 to 14 years. We excluded children aged 6 to 9 years because children’s blood pressure cuffs, which were needed to accurately measure blood pressure, were unavailable for the survey. Women’s and children’s questionnaires contained questions about health status, physical activity, reproductive history (women only), and dietary diversity. Women were asked if they were ever diagnosed with high blood pressure, and if so, whether they were currently taking prescription blood pressure medication.

Anthropometric measures were assessed by following standardized protocols (12). Two trained anthropometrists measured participants’ standing height in cm (without shoes or hairpieces) by using a portable stadiometer (ShorrBoard, Olney, Maryland) and weight in kg (without excess/wet clothing or wet hair, and after using the restroom) using a digital floor scale (Seca 874 Digital Floor Scale, Olney, Maryland). Enumerators measured women’s waist circumference in cm against the skin at the narrowest part of the waistline after exhalation. We calculated women’s body mass index (BMI) by dividing weight (kg) by height (m) squared and categorized it as normal weight/underweight (<25.0 kg/m^2^), overweight (25.0 to <30.0 kg/m^2^), or obesity (≥30.0 kg/m^2^). Women’s waist-to-height ratio was calculated by dividing waist circumference (m) by height (m) and dichotomized as <0.5 or ≥0.5 (13). We calculated children’s BMI-for-age-and-sex *z* score (BMI-*z*) by using the 2007 WHO Growth Reference and categorized BMI-*z* as obesity (>+2 standard deviation [SD]), overweight (>+1 to +2 SD), or normal weight/underweight (≤+1 SD) (14).

Enumerators measured respondents’ blood pressure by using the MDF Lenus Digital Blood Pressure Monitor arm cuff (MDF Instruments, Los Angeles, California) on the left arm after respondents sat for 5 minutes or more. Three blood pressure measurements were taken 1 minute apart. We averaged the second and third measurements (15).

We defined high blood pressure 2 ways among women: 1) using 1999 World Health Organization (WHO) guidelines (16) and 2) using 2017 American College of Cardiology/American Heart Association (ACC/AHA) guidelines (8). WHO defines high blood pressure in adults as systolic blood pressure/diastolic blood pressure of 140/90 mm Hg or more or currently taking blood pressure medication (16). The 2017 ACC/AHA guidelines define high blood pressure as systolic blood pressure/diastolic blood pressure of 130/80 mm Hg or more or currently taking blood pressure medication (8).

We classified high blood pressure 2 ways among children aged 10 to 14 years: 1) using 2004 guidelines from the fourth report of the National Heart, Lung, and Blood Institute (17) and 2) using 2017 American Academy of Pediatrics (AAP) guidelines (18). The 2004 guidelines define hypertension as blood pressure in the 95th percentile or more for age, sex, and height (blood pressure charts were derived from children of all BMI categories) (17). Following the 2017 AAP guidelines, we defined hypertension as blood pressure in the 95th percentile or more for age, sex, and height or blood pressure of 130/80 mm Hg or more (18) (blood pressure charts were derived from children with normal weight only [19]). Although clinicians do not typically use blood pressure percentiles to define hypertension among children aged 13 years or older, blood pressure percentiles may be used to provide more precise surveillance or research estimates (19).

We calculated height-for-age *z* score (HFA-*z*, for calculation of blood pressure percentiles using 2004 guidelines) using the 2007 WHO Growth Reference; we excluded 3 children with an HFA-*z* less than −6 or more than 6 (14). Height in cm was used to derive pediatric blood pressure percentiles using 2017 AAP guidelines (18).

After blood pressure was measured, women’s venous blood samples were collected, the cold chain was maintained, and samples were analyzed at INCAP. Women’s hemoglobin A_1c_ (HbA_1c_) levels were assessed by using Siemens 5035C test kits and the DCA analyzer (Siemens Diagnostics, Erlangen, Germany; National Glycohemoglobin Standardization Program–certified). Diabetes was defined as HbA_1c_ of 6.5% or more (20). Data on use of diabetes medication were not available.

### Statistical analysis

We restricted analyses to participants with complete data and conducted analyses separately by population group. To correctly estimate standard errors, we used a domain statement in SAS survey procedures. Results were exported for participants with complete data.

We calculated the prevalence of high blood pressure overall and by participant characteristics and used χ^2^ tests to evaluate whether high blood pressure prevalence varied by these characteristics. We calculated the prevalence of hypertension awareness (previous diagnosis), treatment, and control (among those treated, blood pressure <140/90 mm Hg [1999 WHO guidelines] or <130/80 mm Hg [2017 ACC/AHA guidelines]) among women. We accounted for SIVESNU’s design effects by using SAS complex survey procedures (SAS version 9.4, Cary, North Carolina). Significance was set at *P* < .05.

We used multivariable logistic regression with backward elimination to identify significant predictors of each outcome. Certain key variables were kept in a base model, regardless of significance, on the basis of evidence in the scientific literature. Among women, these theoretical 5 variables were age category, ethnicity, socioeconomic index, waist-to-height ratio category, and BMI category. We used backward elimination to assess whether other potential predictors should be added to the base model. Candidate predictor variables among women were diabetes, household food security, minimum dietary diversity, smoking history, physical activity, sedentary behavior, household size, urban residence, education, parity (number of pregnancies reaching ≥20 weeks of gestation), and history of hormonal contraceptive use. We evaluated interaction terms between BMI category and both food security and physical activity. In sensitivity analyses, we restricted analyses to women without a previous hypertension diagnosis.

Among children aged 10 to 14 years, 5 variables remained in the model, regardless of significance: sex, age category, ethnicity, socioeconomic index, and BMI-*z* category. Candidate predictor variables in children aged 10 to 14 years were physical activity, sedentary behavior, minimum dietary diversity, household food security, urban residence, household size, education of household head, and interaction terms between BMI-*z* category and physical activity, sedentary behavior, ethnicity, and food security.

### Informed consent

Adults provided oral and written informed consent for themselves and their children, and children aged 10 to 14 years provided assent. The Guatemalan Ministry of Health Institutional Review Board approved the 2017 SIVESNU. We analyzed a de-identified data set. The US Centers for Disease Control and Prevention classified this project as public health practice.

## Results

Community leaders from 96 of 100 enumeration areas agreed to data collection. From 2,880 selected households in 96 enumeration areas, 2,424 households, 1,741 women aged 15 to 49, and 644 children aged 10 to 14 years participated. The response rate was 87.5% among women and 91.8% among children. After exclusion of pregnant women and participants without complete data on all covariates of interest, 1,182 non-pregnant women aged 15 to 49 and 560 children aged 10 to 14 years remained in our sample (Figure). Women with complete data on all covariates had a slightly lower socioeconomic index than women without complete data. Children aged 10 to 14 years with complete data on all covariates were slightly more likely to be girls, aged 10 to 12 years, have a lower BMI, and come from a low-socioeconomic–index household than children without complete data.

**Figure:**
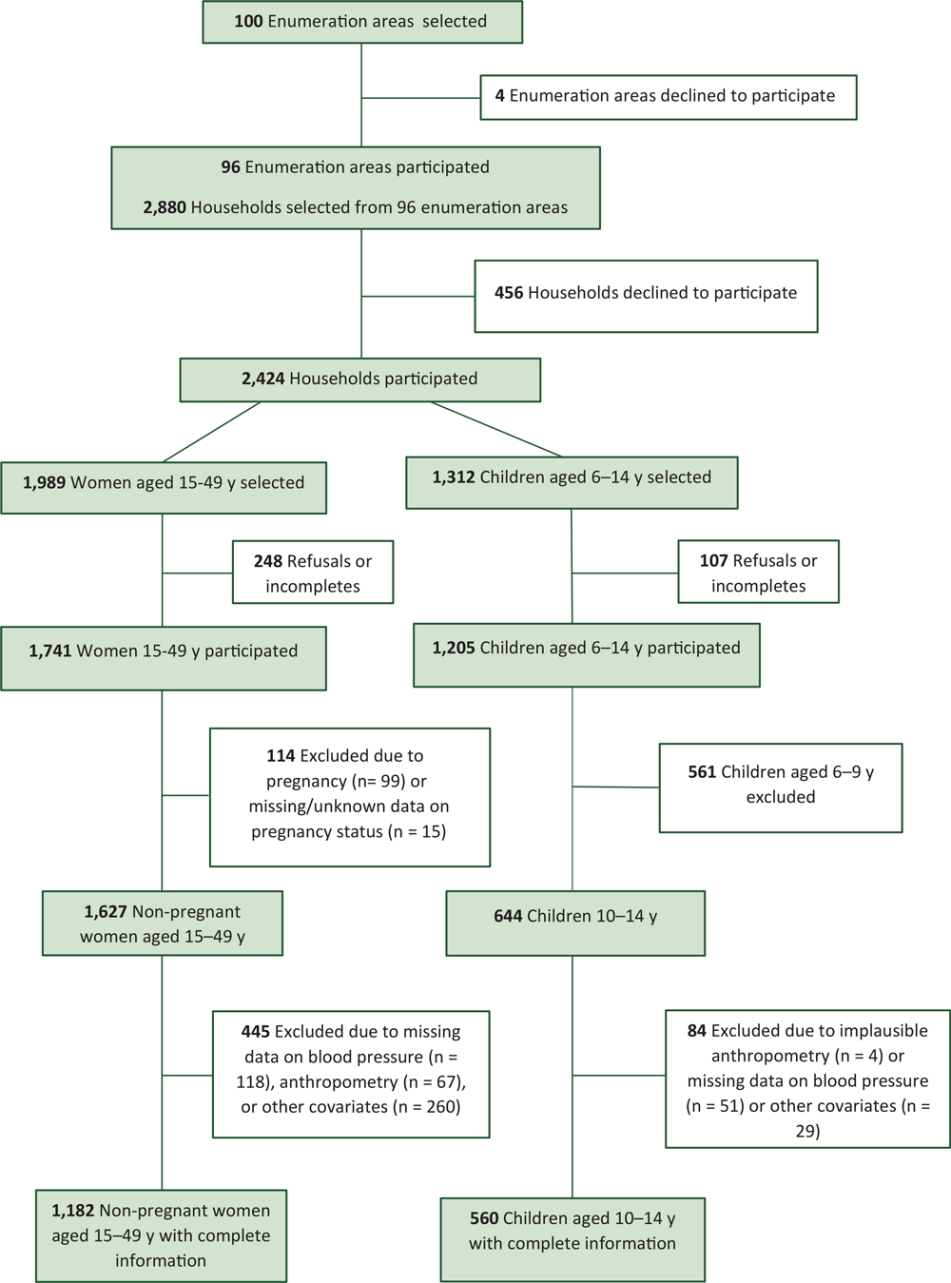
Inclusion and exclusion criteria for a study on the prevalence and predictors of high blood pressure among children aged 10–14 years and non-pregnant women aged 15–49, Guatemala, 2017.

Among children aged 10 to 14 years, the prevalence of high blood pressure was 8.0% (95% confidence interval [CI], 5.4%–10.7%) according to 2004 guidelines and 14.0% (95% CI, 10.6%–17.5%) according to 2017 guidelines (Table 1). In addition, 11.4% of children had prehypertension according to 2004 guidelines, and 10.8% had elevated blood pressure according to 2017 guidelines (Table 2). High blood pressure was more common in children with overweight or obesity and children in food-secure households (Table 1). The prevalence of high blood pressure based on 2017 AAP guidelines was 9.4% among children with normal weight/underweight, 26.4% among children with overweight, and 40.5% among children with obesity (Table 1).

**Table 1:** Prevalence of High Blood Pressure in Guatemalan Children Aged 10–14 Years and Non-Pregnant Women Aged 15–49, 2017^a^

Characteristic	Children Aged 10–14 Years			Non-Pregnant Women Aged 15–49		
	No. in Total Sample	No. (%) With High Blood Pressure, 2004 Guidelines^b^	No. (%) With High Blood Pressure, 2017 Guidelines^c^	No. in Total Sample	No. (%) With High Blood Pressure, 1999 WHO Guidelines^d^	No. (%) With High Blood Pressure, 2017 ACC/AHA Guidelines^e^
**Total, no. (% [95% CI])**	560	45 (8.0 [5.4–10.7])	81 (14.0 [10.6–17.5])	1,182	163 (12.7 [10.7–14.8])	480 (41.1 [37.7–44.4])
**Sex**						
Male	261	18 (6.0)	42 (14.2)	—	—	—
Female	299	27 (9.8)	42 (13.9)	—	—	—
**Age, y**						
10–12	352	30 (8.6)	54 (15.2)	—	—	—
13–14	208	15 (7.0)	27 (11.8)	—	—	—
15–29	—	—	—	588	42 (6.4)^f^	170 (29.1)^f^
30–39	—	—	—	359	53 (14.7)^f^	159 (47.2)^f^
40–49	—	—	—	235	68 (27.3)^f^	151 (65.1)^f^
**Indigenous**						
Yes	246	24 (9.3)	42 (16.7)	530	68 (11.7)	216 (41.6)
No	314	21 (7.0)	39 (11.7)	652	95 (13.6)	264 (40.6)
**Socioeconomic index^g^**						
Low	238	15 (6.3)	32 (13.3)	487	64 (11.9)	196 (42.6)
Medium	209	23 (10.4)	32 (14.5)	463	63 (13.9)	192 (41.6)
High	113	7 (8.0)	17 (14.9)	232	36 (12.4)	92 (36.9)
**Body mass index category^h^**						
Normal weight/underweight	423	19 (4.6)^f^	38 (9.4)^f^	495	29 (5.7)^f^	131 (27.5)^f^
Overweight	86	15 (20.1)^f^	21 (26.4)^f^	378	63 (17.6)^f^	172 (46.7)^f^
Obesity	51	11 (21.6)^f^	22 (40.5)^f^	309	71 (20.0)^f^	177 (59.4)^f^
**Waist-to-height ratio**						
<0.5	—	—	—	146	6 (4.4)^f^	18 (12.0)^f^
≥0.5	—	—	—	1,036	157 (14.1)^f^	462 (45.8)^f^
**Stature**						
Children aged 10–14 y						
Severe stunting^i^	23	1 (2.1)	1 (2.1)	—	—	—
Moderate stunting^i^	128	7 (6.9)	15 (12.7)	—	—	—
No stunting^i^	409	37 (8.8)	65 (15.3)	—	—	—
Non-pregnant women aged 15–49						
Height <150 cm	—	—	—	256	30 (10.4)	101 (41.7)
Height ≥150 cm	—	—	—	926	133 (13.4)	379 (40.9)
**Diabetes**						
Yes	—	—	—	56	23 (39.8)^f^	41 (75.9)^f^
No	—	—	—	1,126	140 (11.4)^f^	439 (39.3)^f^
**Food secure**						
Yes	78	14 (18.8)^f^	19 (26.5)^f^	160	18 (8.6)	73 (45.5)
No	482	31 (6.7)^f^	62 (12.4)^f^	1,022	145 (13.4)	407 (40.4)
**Attained minimum dietary diversity**						
Yes	386	30 (8.0)	53 (13.3)	782	113 (13.1)	73 (45.5)
No	174	15 (8.2)	28 (15.5)	400	50 (12.0)	407 (40.4)
**Ever smoked**						
Yes	—	—	—	89	13 (13.6)	316 (39.4)
No	—	—	—	1,093	150 (12.7)	164 (44.3)
**Physical activity**						
Children aged 10–14 y						
Obtains ≥60 min physical activity daily on ≥5 days/week	154	8 (4.9)	15 (9.0)	—	—	—
Does not obtain ≥60 min physical activity daily on ≥5 days/week	406	37 (9.3)	66 (16.0)	—	—	—
Non-pregnant women aged 15–49						
Meets WHO physical activity recommendations^j^	—	—	—	751	102 (12.2)	296 (38.5)^f^
Does not meet WHO physical activity recommendations^j^	—	—	—	431	61 (13.6)	184 (45.3)^f^
**Sedentary behavior**						
Children aged 10–14 y						
Sits ≥3 h daily, not including schoolwork	426	36 (8.8)	59 (13.7)	—	—	—
Sits <3 h daily, not including schoolwork	134	9 (5.8)	22 (15.1)	—	—	—
Non-pregnant women aged 15–49						
Sits ≥5 h daily, not including sleeping	—	—	—	634	79 (11.3)	225 (35.5)^f^
Sits <5 h daily, not including sleeping	—	—	—	548	84 (14.5)	255 (47.6)^f^
**Household size, no. of members**						
1–3	51	4 (8.8)	8 (15.8)	245	48 (19.1)^f^	98 (39.6)
4–6	331	29 (9.2)	51 (14.3)	669	85 (12.5)^f^	279 (41.5)
≥7	178	12 (6.5)	22 (13.4)	268	30 (10.2)^f^	103 (41.1)
**Residence**						
Urban	192	18 (9.1)	30 (14.2)	397	60 (14.8)	151 (38.4)
Rural	368	27 (7.6)	51 (13.9)	785	103 (11.8)	329 (42.3)
**Head of household ever attended school**						
Yes	442	37 (8.3)	66 (14.1)	976	141 (13.3)	402 (41.5)
No	118	8 (7.3)	15 (13.7)	205	22 (10.2)	78 (39.5)
**Ever attended school^k^**						
Yes	551	45 (8.2)	81 (14.3)	1,023	137 (12.3)	401 (39.9)^f^
No	5	0 (0.0)	0 (0.0)	159	26 (16.2)	79 (50.0)^f^
**Parity**						
0	—	—	—	299	31 (8.4)^f^	94 (32.5)^f^
1-4	—	—	—	712	90 (12.0)^f^	299 (42.6)^f^
≥5	—	—	—	171	42 (24.9)^f^	87 (55.1)^f^
**Ever use of hormonal contraception**						
Yes	—	—	—	582	83 (15.2)^f^	248 (43.9)
No	—	—	—	600	80 (11.0)^f^	232 (38.9)
**Current use of hormonal contraception^l^**						
Yes	—	—	—	199	21 (9.7)	77 (36.8)
No	—	—	—	848	114 (12.1)	343 (41.2)

Abbreviations: —, does not apply; ACC, American College of Cardiology; AHA, American Heart Association; BMI, body mass index; WHO, World Health Organization.

a Data source: 2017 Sistema de Vigilancia Epidemiológica de Salud y Nutrición (SIVESNU) (Epidemiological Health and Nutrition Surveillance System) (10). Sample sizes are unweighted, but prevalences are weighted. Values are number (percentage) unless otherwise indicated.

b Systolic blood pressure or diastolic blood pressure ≥95th percentile for age, sex, and height (blood pressure charts derived from children in all BMI categories) (17).

c Systolic blood pressure or diastolic blood pressure ≥95th percentile for age, sex, and height, systolic blood pressure ≥130 mm Hg, or diastolic blood pressure ≥80 mm Hg (blood pressure charts derived from children with normal BMI) (18).

d Systolic blood pressure ≥140 mm Hg, diastolic blood pressure ≥90 mm Hg, or taking blood pressure medication (16).

e Systolic blood pressure ≥130 mm Hg, diastolic blood pressure ≥80 mm Hg, or taking blood pressure medication (8).

f Indicates *P* < .05 for an *F* statistic evaluating the null hypothesis of no association between each covariate and high blood pressure; significance set at *P* < .05. Hypothesis tests were conducted separately for non-pregnant women of reproductive age and children aged 10–14 years.

g We derived a socioeconomic index variable using principal components analysis with 5 household-level input variables (radio, television, toilet, exclusive cooking area, and handwashing soap). The largest principal component was ranked into 5 groups. Low socioeconomic index was defined as a score of 0 or 1, medium socioeconomic index as a score of 2 or 3, and high socioeconomic index as a score of 4.

h Among children aged 10–14 years: normal weight/underweight was defined as body mass index-for-age-and-sex *z* score (BMI-*z*) <+1 standard deviation (SD); overweight, BMI-*z* > +1 to +2 SD; and obesity, BMI-*z* >+2 SD. Among non-pregnant women aged 15–49, normal weight/underweight was defined as BMI <25.0 kg/m^2^; overweight, BMI 25.0 to <30.0 kg/m^2^; obesity, BMI ≥30.0 kg/m^2^.

i No stunting, height-for-age *z* score (HFA-*z*) ≥ −2; moderate stunting, HFA-*z* ≥ −3 to <−2; severe stunting, HFA-*z* <−3.

j Defined as ≥75 min of vigorous activity weekly, ≥150 min moderate activity weekly, or an equivalent combination of the two (21).

k 4 children were missing data on education.

l 1,047 women had data on current contraceptive use.

**Table 2:** Prevalence of Hypertension, Prehypertension, Elevated Blood Pressure, and Hypertension Awareness (Diagnosis), Treatment, and Control in Guatemalan Children 10–14 Years and Non-Pregnant Women 15–49 Years, 2017^a^

Characteristic	Children Aged 10–14 Years (n = 560)		Non-Pregnant Women Aged 15−49 (n = 1,182)	
	No. (%) Using 2004 Guidelines^b^	No. (%) Using 2017 Guidelines^c^	No. (%) Using 1999 WHO Guidelines^d^	No. (%) Using 2017 ACC/AHA Guidelines^e^
**Has hypertension **	45 (8.0)	81 (14.0)	163 (12.7)	480 (41.1)
**Has prehypertension **	66 (11.4)	—f	368 (32.4)	—f
**Has elevated blood pressure**	—f	59 (10.8)	—f	51 (4.1)
**Has normal blood pressure**	449 (80.6)	420 (75.2)	651 (54.8)	651 (54.8)
**Among women with hypertension**				
Awareness (ever diagnosed)	—	—	72 (45.7)	133 (28.2)
Using prescription blood pressure medication^g^	—	—	38 (26.0)^g^	38 (8.0)^g^
**Among women with a previous diagnosis of hypertension **				
Using prescription blood pressure medication^g^	—	—	38 (58.2)^g^	38 (29.4)^g^
**Among women using prescription blood pressure medication**				
Hypertension is controlled^h^	—	—	23 (56.1)^h^	10 (26.3)^h^

Abbreviations: —, does not apply; ACC, American College of Cardiology; AHA, American Heart Association; BMI, body mass index; WHO, World Health Organization.

a Data source: 2017 Sistema de Vigilancia Epidemiológica de Salud y Nutrición (SIVESNU) (Epidemiological Health and Nutrition Surveillance System) (10). Sample sizes are unweighted; prevalences are weighted.

b Blood pressure charts were derived from children with all body mass categories. Hypertension was defined as systolic blood pressure or diastolic blood pressure ≥95th percentile for age, sex, and height. Prehypertension was defined as systolic blood pressure or ≥90th to <95th percentile, systolic blood pressure ≥120 mm Hg to <95th percentile, or diastolic blood pressure ≥80 mm Hg to <95th percentile. Normal blood pressure was defined as systolic blood pressure and diastolic blood pressure <90th percentile (17).

c Blood pressure charts were derived from children with normal body mass index. Hypertension was defined as systolic blood pressure or diastolic blood pressure ≥95th percentile for age, sex, and height, systolic blood pressure ≥130 mm Hg, or diastolic blood pressure ≥80 mm Hg. Elevated blood pressure was defined as systolic blood pressure or diastolic blood pressure ≥90th to <95th percentile or systolic blood pressure ≥120 mm Hg to <95th percentile. Normal blood pressure was defined as systolic blood pressure and diastolic blood pressure ≤90th percentile (18).

d Hypertension was defined as systolic blood pressure ≥140 mm Hg, diastolic blood pressure ≥90 mm Hg, or using prescription blood pressure medication. Prehypertension was defined as systolic blood pressure 120 to <140 mm Hg or diastolic blood pressure 80 to <90 mm Hg (and not using prescription blood pressure medication). Normal blood pressure was defined as systolic blood pressure <120 mm Hg, diastolic blood pressure <80 mm Hg, and not using prescription blood pressure medication (16).

e Hypertension was defined as systolic blood pressure ≥130 mm Hg, diastolic blood pressure ≥80 mm Hg, or using prescription blood pressure medication. Elevated blood pressure was defined as systolic blood pressure 120 to <130 mm Hg and diastolic blood pressure <80 mm Hg (and not using prescription blood pressure medication). Normal blood pressure was defined as systolic blood pressure <120 mm Hg, diastolic blood pressure <80 mm Hg, and not using prescription blood pressure medication (8).

f Prehypertension was a defined blood pressure category in the 1999 WHO guidelines (16) and 2004 pediatric guidelines (17) but not in the 2017 ACC/AHA guidelines (8) nor the 2017 AAP guidelines (18). Elevated blood pressure was a defined blood pressure category in the 2017 ACC/AHA and 2017 AAP guidelines only (8,18).

g Although the 2017 ACC/AHA guidelines defined high blood pressure as 130/80 mm Hg, the cutoffs for initiating pharmacologic treatment for most people is 140/90 mm Hg (8).

h According to 1999 WHO guidelines (16), controlled hypertension is defined as systolic blood pressure <140 mm Hg/diastolic blood pressure <90 mm Hg among those being treated. According to 2017 ACC/AHA guidelines (8), controlled hypertension is defined as systolic blood pressure <130/diastolic blood pressure <80 mm Hg among those being treated.

Among women, the prevalence of high blood pressure was 12.7% (95% CI, 10.7%–14.8%) according to 1999 WHO guidelines and 41.1% (95% CI, 37.7%–44.4%) according to 2017 ACC/AHA guidelines (Table 1). In addition, 32.4% had prehypertension according to 1999 WHO guidelines, and 4.1% had elevated blood pressure according to 2017 ACC/AHA guidelines (Table 2). High blood pressure was more common among women with older age, overweight or obesity, a waist-to-height ratio of 0.5 or more, diabetes, and high parity according to both guidelines (Table 1). Based on 2017 ACC/AHA guidelines, the prevalence of high blood pressure was more than twice as high among women with obesity (59.4%) as among women with normal weight/underweight (27.5%) and nearly twice as high among women with diabetes (75.9%) as among women without diabetes (39.3%) (Table 1). According to 1999 WHO guidelines only, high blood pressure was more common among women with a small household size (*P* = .02) and among women who ever used hormonal contraception (*P* *= *.03) (Table 1). According to 2017 ACC/AHA guidelines only, high blood pressure was more common among women who did not meet WHO physical activity recommendations (*P* = .02), who sat fewer than 5 hours per day (*P* <.001), and who never attended school (*P* = .03) (Table 1).

The prevalences of awareness, treatment, and control of high blood pressure were low among reproductive-aged women. Less than half of non-pregnant women aged 15 to 49 with hypertension had a previous diagnosis of hypertension (1999 WHO guidelines, 45.7%; 2017 ACC/AHA guidelines, 28.2%), and less than 30% of women with hypertension were using prescription blood pressure medication (Table 2). Less than 60% of women using blood pressure medication had their blood pressure controlled (1999 WHO guidelines, 56.1%; 2017 ACC/AHA guidelines: 26.3%).

The final multivariable models for children aged 10 to 14 years included only the 5 core variables in the base model: sex, age category, ethnicity, socioeconomic index, and BMI-*z* category. Other predictors were excluded during backward elimination (Table 3). Results were similar by high blood pressure definition. The odds of high blood pressure were twice as high among indigenous children than among nonindigenous children (adjusted odds ratios [ORs] = 2.14 [95% CI, 1.13–4.05] according to 2004 guidelines and 2.20 [95% CI, 1.29–3.77] according to 2017 AAP guidelines). Compared with children with normal weight/underweight, children with overweight had 6.10 (95% CI, 3.03–12.29) times higher odds of high blood pressure according to 2004 guidelines and 4.42 (95% CI, 1.98–9.86) times higher odds of high blood pressure according to 2017 guidelines. Associations were stronger for obesity and high blood pressure: children with obesity had 7.10 (95% CI, 2.96–17.03) times higher odds of high blood pressure according to 2004 guidelines and 9.14 (95% CI, 4.14–20.20) times higher odds of high blood pressure according to 2017 guidelines compared with children with normal weight/underweight (Table 3).

**Table 3:** Predictors of Hypertension Among Guatemalan Children Aged 10–14 Years, 2017^a^

Characteristic	Using 2004 High Blood Pressure Guidelines^b^	Using 2017 High Blood Pressure Guidelines^c^
**Sex**		
Girl	1.79 (0.79–4.02) [.16]	1.00 (0.58–1.72) [.99]
Boy	1 [Reference]	1 [Reference]
**Age, y**		
10–12	1 [Reference]	1 [Reference]
13–14	0.91 (0.43–1.93) [.81]	0.83 (0.49–1.40) [.48]
**Indigenous**		
Yes	2.14 (1.13–4.05) [.02]	2.20 (1.29–3.77) [.004]
No	1 [Reference]	1 [Reference]
**Socioeconomic index^d^**		
Low	1.12 (0.39–3.19) [.83]	1.31 (0.63–2.72) [.47]
Medium	1.55 (0.58–4.11) [.37]	1.08 (0.57–2.07) [.81]
High	1 [Reference]	1 [Reference]
**Body mass index category^e^**		
Obesity	7.10 (2.96–17.03) [<.001]	9.14 (4.14–20.20) [<.001]
Overweight	6.10 (3.03–12.29) [<.001]	4.42 (1.98–9.86) [<.001]
Normal weight/underweight	1 [Reference]	1 [Reference]

a All values are adjusted odds ratio (95% confidence interval) [*P* value]. Key covariates that remained in the model regardless of *P* value included sex (boy/girl), age category (10–12 vs 13–14 y), self-reported indigenous status (yes/no), socioeconomic index (low, medium, high), and BMI *z* category (obesity, overweight, normal weight/underweight). Potential predictor variables evaluated through backward elimination included obtaining ≥60 min of physical activity on ≥5 days per week (yes/no), sitting ≥3 h daily outside of schoolwork (yes/no), sufficient dietary diversity (yes/no), household food security (secure/not secure), residence (urban/rural), household size (1-3, 4–6, 7–8, ≥9 members), education of head of household (some/none), an interaction term between BMI *z* category and physical activity, an interaction term between BMI *z* category and sedentary behavior, an interaction term between BMI *z* category and indigenous status, and an interaction term between BMI *z* category and food security. Data source: 2017 Sistema de Vigilancia Epidemiológica de Salud y Nutrición (SIVESNU) (Epidemiological Health and Nutrition Surveillance System) (10).

b Systolic blood pressure or diastolic blood pressure ≥95th percentile; blood pressure charts were derived from children in all categories of body mass index (17).

c Systolic blood pressure or diastolic blood pressure ≥95th percentile; systolic blood pressure ≥130 mm Hg, or diastolic blood pressure ≥80 mm Hg; blood pressure charts were derived from children with normal body mass index only (18).

d We derived a socioeconomic index variable using principal components analysis with 5 household-level input variables. The largest principal component was ranked into 5 groups. Low socioeconomic index, score of 0 or 1; medium socioeconomic index, score of 2 or 3; high socioeconomic index, score of 4.

e Normal weight/underweight was defined as body mass index-for-age-and-sex z score (BMI-*z*) <+1 standard deviation (SD); overweight, BMI-z, >+1 to +2 SD, and obesity, BMI-*z*, >+2 SD.

The final predictors remaining in multivariable models among women differed slightly by high blood pressure definition. Using 1999 WHO high blood pressure guidelines, the final multivariable model for women included 5 core variables from the base model (age category, ethnicity, socioeconomic index, waist-to-height ratio category, and BMI category) plus diabetes, parity, household size, and food security (Table 4). The final model using 2017 ACC/AHA high blood pressure guidelines included the 5 core variables and diabetes. Increasing age category predicted high blood pressure according to both guidelines (adjusted OR [95% CI] for age 40–49 vs age 15–29: 2.87 [1.63–5.05] according to 1999 WHO guidelines and 2.95 [2.00–4.35] according to 2017 ACC/AHA guidelines). Regardless of high blood pressure definition, women with obesity had approximately twice the odds of high blood pressure than women with normal weight/underweight (adjusted ORs were 2.33 [95% CI, 1.22–4.42] according to 1999 WHO guidelines and 2.04 [95% CI, 1.38–3.03] using 2017 ACC/AHA guidelines). Overweight was associated with increases in the odds of having high blood pressure according to the 1999 WHO definition only (adjusted OR = 2.41; 95% CI, 1.38–4.21). Additionally, the odds of high blood pressure were twice as high among women with diabetes (HbA_1c_ ≥6.5%) than among women without diabetes, regardless of high blood pressure definition (eg, adjusted OR = 2.09 [95% CI, 1.07–4.07] according to 2017 ACC/AHA guidelines). Waist-to-height ratio category was a core variable retained in both final models; however, it was associated with increases in the odds of having high blood pressure according to the 2017 ACC/AHA high blood pressure definition only (adjusted OR for waist-to-height ratio ≥0.5 vs <0.5: 3.43 [95% CI, 1.74–6.77]; Table 4). When following 1999 WHO guidelines only, the odds of high blood pressure were higher among women in food-insecure (vs secure) households (adjusted OR 1.81 [95% CI, 1.06–3.09]) but lower in households with more than 3 (vs 3 or fewer) people (Table 4). In sensitivity analyses restricted to women never diagnosed with high blood pressure, most results were unchanged; however, diabetes, parity, and food security were no longer associated with high blood pressure.

**Table 4:** Predictors of Hypertension Among Non-Pregnant Guatemalan Women Aged 15–49, 2017^a^

Characteristic	Using 1999 WHO High Blood Pressure Guidelines^b^	Using 2017 ACC/AHA High Blood Pressure Guidelines^c^
**Age, y**		
15–29	1 [Reference]	1 [Reference]
30–39	1.94 (1.16–3.26) [.01]	1.58 (1.12–2.24) [.01]
40–49	2.87 (1.63–5.05) [<.001]	2.95 (2.00–4.35) [<.001]
**Indigenous**		
Yes	0.87 (0.56–1.36) [.54]	1.06 (0.80–1.41) [.69]
No	1 [Reference]	1 [Reference]
**Socioeconomic index^d^**		
Low	0.98 (0.57–1.66) [.93]	1.45 (0.92–2.30) [.11]
Medium	1.02 (0.61–1.68) [.95]	1.19 (0.77–1.84) [.42]
High	1 [Reference]	1 [Reference]
**Body mass index category^e^**		
Obesity	2.33 (1.22–4.42) [.01]	2.04 (1.38–3.03) [<.001]
Overweight	2.41 (1.38–4.21) [.002]	1.35 (0.94–1.93) [.10]
Normal weight/underweight	1 [Reference]	1 [Reference]
**Waist-to-height ratio**		
≥0.5	1.47 (0.37–5.81) [.58]	3.43 (1.74–6.77) [<.001]
<0.5	1 [Reference]	1 [Reference]
**Diabetes**		
Yes	2.40 (1.19–4.85) [.02]	2.09 (1.07–4.07) [.03]
No	1 [Reference]	1 [Reference]
**Parity**		
≥5	1.22 (0.56–2.66) [.61]	—
1–4	0.65 (0.38–1.09) [.10]	—
0	1 [Reference]	—
**Household size**		
≥7	0.42 (0.22–0.82) [.01]	—
4–6	0.54 (0.35–0.83) [.01]	—
1–3	1 [Reference]	—
**Food secure**		
Yes	1 [Reference]	—
No	1.81 (1.06–3.09) [.03]	—

Abbreviations: — , not included in model; ACC, American College of Cardiology; AHA, American Heart Association; BMI, body mass index; WHO, World Health Organization.

a All values are adjusted odds ratio (95% confidence interval) [*P* value]. Key variables were kept in the model regardless of significance. These key variables included age category, indigenous ethnicity, socioeconomic index, waist-to-height ratio ≥0.5, and BMI category. Additional women’s predictor variables that were evaluated through backward elimination included: diabetes (yes /no), household food security (yes/no), sufficient dietary diversity (Minimum Dietary Diversity Score for Women ≥5 of 10), self-reported smoking history (ever/never smoked), physical activity (meets or does not meet current World Health Organization physical activity guidelines of ≥75 min of vigorous activity weekly, ≥150 min moderate activity weekly, or an equivalent combination of the two), sedentary behavior (<5 vs ≥5 h/d [<median vs ≥median]), household size (1-3, 4–6, ≥7 members), area of residence (urban/rural), education (ever/never attended school), parity (number of pregnancies reaching ≥20 weeks of gestation; categorized as 0, 1–4, or ≥5), and ever use of hormonal contraception (yes/no). We also evaluated interaction terms between BMI category and both food security and physical activity. Data source: 2017 Sistema de Vigilancia Epidemiológica de Salud y Nutrición (SIVESNU) (Epidemiological Health and Nutrition Surveillance System) (10).

b Systolic blood pressure ≥140 mm Hg, diastolic blood pressure ≥90 mm Hg, or on blood pressure medication (16).

c Systolic blood pressure ≥130 mm Hg, diastolic blood pressure ≥80 mm Hg, or on blood pressure medication (8).

d We derived a socioeconomic index variable using principal components analysis with 5 household-level input variables. The largest principal component was ranked into 5 groups. Low socioeconomic index, score of 0 or 1; medium socioeconomic index, score of 2 or 3; high socioeconomic index, score of 4.

e Normal weight/underweight was defined as BMI <25.0 kg/m^2^; overweight, BMI 25.0 to <30.0 kg/m^2^; obesity, BMI ≥30.0 kg/m^2^.

## Discussion

In this nationally representative study, high blood pressure was common among children aged 10 to 14 years and non-pregnant women aged 15 to 49 in Guatemala, but prevalence varied considerably by high blood pressure guideline. The prevalence of high blood pressure among children was nearly twice as high when the 2017 pediatric guidelines were used than when the 2004 pediatric guidelines were used. Similarly, high blood pressure prevalence was more than 3 times higher among women when the 2017 ACC/AHA guidelines were used than when the 1999 WHO guidelines were used. The prevalences of hypertension awareness, treatment, and control were low among non-pregnant women aged 15 to 49 in Guatemala.

Most findings from multivariable models were similar by high blood pressure definition. As hypothesized, overweight and obesity were strong risk factors for high blood pressure in both demographic groups. These associations mirror trends reported in other studies (7,8) and are concerning given the high prevalence of overweight and obesity in Guatemala. The prevalence of high blood pressure increased with age among women, an association documented elsewhere (8). In addition, the odds of high blood pressure were twice as high among women with diabetes as among women without diabetes. Diabetes may increase the risks and complications of high blood pressure (22). Although indigenous children were less likely than nonindigenous children to have obesity and other risk factors for high blood pressure, they had higher odds of high blood pressure in adjusted models. Future studies should explore these differences in detail.

Nationally representative data on high blood pressure among Latin American children are sparse. According to 2004 pediatric guidelines, high blood pressure prevalence among children aged 10 to 14 years in our study (8.0%) was higher than the 6.2% pooled prevalence among Latin American children and adolescents aged 10 to 19 years (14 studies, none from Central America) but lower than the 9.8% pooled prevalence among adolescents from low- and middle-income countries in a recent systematic review (4). A small number of published studies assessed high blood pressure among children using 2017 AAP guidelines. Similar to our analysis, a nationally representative study of US adolescents aged 12 to 17 in 2013–2016 found higher high blood pressure prevalence when 2017 guidelines were used than when 2004 guidelines were used (3.2% vs 1.6%, respectively) (7).

Evidence on high blood pressure among non-pregnant women aged 15 to 49 in Latin America is scant, and most Latin American studies are not nationally representative. Using 1999 WHO cutoffs, the 12.7% high blood pressure prevalence among Guatemalan women in our study was slightly lower than in Paraguayan women in 2011 (6) and women in low- and middle-income countries in 2010 (23), while it was higher than the 2015 estimated prevalences of hypertension among reproductive-aged women in Latin America/the Caribbean (ranging from 1.4% among women aged 18–19 to 19.4% among women aged 45–49) and globally (24). Similar to the results of our study, less than half of women with hypertension in low- and middle-income countries were aware of their condition in 2010, and less than one-third had their high blood pressure controlled according to 1999 WHO guidelines (23). Only a small number of studies assessed high blood pressure using 2017 ACC/AHA guidelines. In Peruvian women of all ages, high blood pressure prevalence was twice as high when the ≥130/80 cutoff was used than when the ≥140/90 cutoff was used (25).

The 2017 ACC/AHA and AAP high blood pressure guidelines have advantages over previous high blood pressure definitions, because they are based on current epidemiologic evidence. The 2017 ACC/AHA guidelines reduced adult blood pressure cutoffs for hypertension as a result of evidence of increased cardiovascular risk between 130 to 139 mm Hg systolic pressure and 80 to 89 mm Hg diastolic pressure (8). However, some questions remain about the applicability of new blood pressure treatment thresholds to patients younger than 45 (26). The 2004 pediatric blood pressure percentiles were derived from children of all BMI categories, including children with obesity (17). Consequently, the 2004 blood pressure cutoffs may be biased upward. This bias would underestimate high blood pressure prevalence compared with the 2017 guidelines, which are based on children with normal weight (19). We used traditional cutoffs for high blood pressure in our study because these are used in Guatemala. The choice of cutpoint has broader implications in referral patterns and treatment, which should be balanced with Guatemala’s capacity and resources.

Improving access to hypertension screening and treatment is vital to improving detection and control of high blood pressure. However, health care access is a challenge for many Guatemalans. Assessing blood pressure in community settings could improve hypertension awareness (27). The Pan American Health Organization (PAHO) is working to increase availability and affordability of antihypertensive drugs (28). Both the 1999 WHO and 2017 ACC/AHA guidelines recommend pharmacologic treatment of adults with blood pressure of 140/90 mm Hg or more, although treatment goals differ (<140/90 vs <130/80, respectively) (8,16). Additionally, the 2017 guidelines recommend pharmacologic treatment of adults with blood pressure 130 to 139/80 to 89 mm Hg and elevated cardiovascular risk (ie, clinical cardiovascular disease or an estimated ≥10% ten-year risk of developing atherosclerotic cardiovascular disease); they recommend nonpharmacologic treatment of other adults with blood pressure 130 to 139/80 to 89 mm Hg (8). 

Although more research is needed, our study suggests that almost 30% of Guatemalan women aged 15 to 49 are indicated for nonpharmacologic treatment according to 2017 ACC/AHA guidelines (based on blood pressure 130 to 139/80 to 89 mm Hg), and approximately 13% are indicated for pharmacologic treatment according to either guideline (8,16). Resources to monitor patients receiving blood pressure treatment are limited in Latin America (27). If following 2017 ACC/AHA guidance, it could be prudent to prioritize pharmacologic treatment of women with blood pressure of 140/90 mm Hg or more and to expand treatment services as resources permit.

Both pediatric high blood pressure guidelines recommend lifestyle modifications as a first-line treatment for most children with hypertension (17,18). Given current resource constraints, it may be practical to prioritize treatment of those 8% of children aged 10 to 14 in Guatemala classified as having hypertension based on 2004 guidelines. However, study findings using both the 2017 pediatric and adult guidelines demonstrate that hypertension is more common in Guatemala than previously believed. Regardless of guideline, overweight and obesity were key risk factors for high blood pressure.

The number of Guatemalans with high blood pressure is expected to increase because of population growth and aging (24). Primary prevention of high blood pressure may improve population health and conserve resources (27). PAHO recommends regulating sodium content of foods to reduce high blood pressure prevalence (28). In addition, PAHO endorses population-based obesity prevention strategies, including ensuring health care availability, improving food pricing and labeling, promoting physical activity in workplaces and schools, improving school feeding, and regulating food marketing (29). PAHO also recommends that countries develop multisectoral prevention policies for noncommunicable diseases (28). Guatemala has a strategic plan for preventing obesity and noncommunicable diseases, which is not multisectoral. Furthermore, Guatemala’s National Policy for Food Security and Nutrition focuses primarily on undernutrition (30), and most of Guatemala’s healthy food environment plans are minimally implemented (31). Guatemalan stakeholders recommend creating municipal plans that facilitate implementation of national plans (31).

Our study has limitations. The analysis was cross-sectional, so causality cannot be determined. Digital blood pressure monitors, compared with manual auscultation, can overestimate blood pressure (18). National survey data do not replace clinical measurements. We used a single blood pressure cuff size for all women and children. However, using an adult cuff could underestimate children’s blood pressure (17). We lacked data on children’s blood pressure medication, women’s diabetes medication, and sodium intake (22). Self-reported physical activity data could be misclassified. Finally, results may not be generalizable outside Guatemala. Strengths of our study include timely, representative data on chronic diseases and risk factors among Guatemalan women and children. To our knowledge, ours is the first nationally representative analysis of high blood pressure among Guatemalan women aged 15 to 49 and children aged 10 to 14 and is one of the first to assess high blood pressure prevalence, treatment, and control in Central America using revised definitions of high blood pressure.

In summary, high blood pressure prevalence was high among Guatemalan women aged 15 to 49 and children aged 10 to 14 years, while hypertension awareness, treatment, and control were low among Guatemalan women. Overweight and obesity were strong modifiable risk factors for hypertension in both groups. Expanding access to blood pressure screening and treatment could improve blood pressure awareness and control in Guatemala. Furthermore, obesity prevention and control programs might help prevent high blood pressure in Guatemalan women and children.
